# Supplementary material for: Weak, Broken, but Working—Intramolecular Hydrogen Bond in 2,2′-bipyridine
Source: Int J Mol Sci. 2023 Jun 20;24(12):10390. doi: 10.3390/ijms241210390 (PMC10299186; doi:10.3390/ijms241210390)
Supplement: Supplementary file 1 [file ijms-24-10390-s001.zip › ijms-2462333-supplementary.pdf]

International Journal of *Molecular Sciences*

MDPI

Supporting information

Title

Weak, broken, but working - intramolecular hydrogen bond in 2,2′-bipyridine

Ilya G. Shenderovich

[1]Institute of Organic Chemistry, University of Regensburg, 93053 Regensburg, Germany; ilya.shenderovich@ur.de

Content		Page
Table S1.	The distance r_3, the electronic energy, and the Mulliken charges of the binding proton and the N^f nitrogen of $PyHPy^+$ obtained for selected distances r_2 at ωB97XD/def2tzvp and SCRF=(Solvent=water.	2
Table S2.	The distance r_3, the electronic energy, and the Mulliken charges of the binding proton and the N^f nitrogen of $PyHPy^+$ obtained for selected distances r_2 at TPSSh/def2qzvp and SCRF=(Solvent=water.	2
Table S3.	The distance r_3, the electronic energy, and the Mulliken charges of the binding proton and the N^f nitrogen of $PyHPy^+$ obtained for selected distances r_2 at ωB97XD/def2qzvp and SCRF=(Solvent=water.	3
Table S4.	Geometry of $BiPyH^+$ obtained for selected r_1 at ωB97XD/def2tzvp.	3
Table S5.	Geometry of $PhenH^+$ obtained for selected r_1 at ωB97XD/def2tzvp.	3
Table S6.	Geometry of $BiPyH^+$ obtained for selected r_1 at ωB97XD/def2tzvp SCRF=(Solvent=water).	4
Table S7.	Geometry of $PhenH^+$ obtained for selected r_1 at ωB97XD/def2tzvp SCRF=(Solvent=water).	4
Table S8.	The geometry, energy and N-H stretches of $BiPyH^+$ obtained for selected r_3 at ωB97XD/def2tzvp SCRF=(Solvent=water).	4
Table S9.	Crystallographic data for the single crystal XRD structure of $BiPyH^+$ $[ClO_4]^-$ obtained from acetonitrile.	5
Table S10.	Crystallographic data for the single crystal XRD structure of $BiPyH^+$ $[ClO_4]^-$ obtained from nitromethane.	6
Table S11	The atomic coordinates of $BiPyHClO_4$_Tq_W	7
Table S12	The atomic coordinates of ZUTDAT_Tq_W	7
Table S13	The atomic coordinates of ZUTDAT01_Tq_W	7
Table S14	The atomic coordinates of OPENAN_Tt_W	8
Table S15	The atomic coordinates of OPENAN01_Tt_W	9
Table S16	The atomic coordinates of ZUTDEX_Tq_W	9

Table S1. The N^p…N^f distance (r_3), the electronic energy, and the Mulliken charges of the binding proton ($q(H)$) and the N^f nitrogen ($q(N^f)$) of the hydrogen bonded pyridine-N^p-H…N^f-pyridine cation obtained for selected H…N^f distances (r_2) at ωB97XD/Def2tzvp and SCRF=(Solvent=water.

Substance	r_2 (H...N^f, Å)	r_3 (N^p...N^f, Å)	$q(H)$ (e)	$q(N^f)$ (e)	ε [a] (e^2/Å)	E Hartree
pyridine						−248.288892
pyridinium						−248.7409453
$[PyHPy]^+$	4.987853	6.0	−0.325875	0.321172	−0.02098	−497.0301608
$[PyHPy]^+$	3.987671	5.0	−0.331334	0.317988	−0.02642	−497.0306336
$[PyHPy]^+$	2.983066	4.0	−0.335668	0.303313	−0.03413	−497.0330703
$[PyHPy]^+$	2.780714	3.8	−0.341726	0.294080	−0.03614	−497.0345875
$[PyHPy]^+$	2.576773	3.6	−0.351196	0.284958	−0.03884	−497.0366668
$[PyHPy]^+$	2.474484	3.5	−0.357204	0.280913	−0.04055	−497.0379185
$[PyHPy]^+$	2.268262	3.3	−0.370241	0.276484	−0.04513	−497.0408395
$[PyHPy]^+$	2.059514	3.1	−0.380134	0.273908	−0.05056	−497.0441034
$[PyHPy]^+$	1.953751	3.0	−0.381079	0.272330	−0.05312	−497.0456908
$[PyHPy]^+$	1.900257	2.95	−0.380156	0.271149	−0.05276	−497.04642
$[PyHPy]^+$	1.846680	2.9	−0.378500	0.269806	−0.05374	−497.0470629
$[PyHPy]^+$	1.792227	2.85	−0.375775	0.268202	−0.05458	−497.0475965
$[PyHPy]^+$	1.736974	2.8	−0.372025	0.267112	−0.05545	−497.0479947
$[PyHPy]^+$	1.669100	2.74	−0.365609	0.266495	−0.05609	−497.0482533
$[PyHPy]^+$	1.645433	2.719497	−0.362929	0.266527	−0.05795	−497.0482728
$[PyHPy]^+$	1.523208	2.618	−0.344976	0.268699	−0.06085	−497.0476784

[a]The electric potential energy $\varepsilon = {q(H)q(N^f)}/{r_2}$ associated with the N^f…H interaction.

Table S2. The N^p…N^f distance (r_3), the electronic energy, and the Mulliken charges of the binding proton ($q(H)$) and the N^f nitrogen ($q(N^f)$) of the hydrogen bonded pyridine-N^p-H…N^f-pyridine cation obtained for selected H…N^f distances (r_2) at TPSSh/Def2qzvp and SCRF=(Solvent=water.

Substance	r_2 (H...N^f, Å)	r_3 (N^p...N^f, Å)	$q(H)$ (e)	$q(N^f)$ (e)	ε [a] (e^2/Å)	E Hartree
pyridine						−248.4101725
pyridinium						−248.8613039
$[PyHPy]^+$	2.676394	3.7	−0.372141	0.288767	−0.04015	−497.2758706
$[PyHPy]^+$	2.574324	3.6	−0.369204	0.288999	−0.04145	−497.2768722
$[PyHPy]^+$	2.471759	3.5	−0.366007	0.287572	−0.04258	−497.2780031
$[PyHPy]^+$	2.368570	3.4	−0.362293	0.285675	−0.04370	−497.2792 857
$[PyHPy]^+$	2.264660	3.3	−0.358001	0.284147	−0.04492	−497.2806984
$[PyHPy]^+$	2.159991	3.2	−0.353510	0.283719	−0.04643	−497.2822254

[a]The electric potential energy $\varepsilon = {q(H)q(N^f)}/{r_2}$ associated with the N^f…H interaction.

Table S3. The Np…N^f distance (r_3), the electronic energy, and the Mulliken charges of the binding proton ($q(H)$) and the N^f nitrogen ($q(N^f)$) of the hydrogen bonded pyridine-N^p-H…N^f-pyridine cation obtained for selected H…N^f distances (r_2) at ωB97XD/Def2qzvp and SCRF=(Solvent=water.

Substance	r_2 (H...N^f, Å)	r_3 (N^p...N^f, Å)	$q(H)$ (e)	$q(N^f)$ (e)	ε [a] (e^2/Å)	E Hartree
pyridine						−248.3048658
pyridinium						−248.7574992
[PyHPy]$^+$	2.680144	3.7	0.284131	−0.434869	−0.04610	-497.0676899
[PyHPy]$^+$	2.578196	3.6	0.280033	−0.435436	−0.04730	−497.0688081
[PyHPy]$^+$	2.475891	3.5	0.275616	−0.436552	−0.04860	-497.0700663
[PyHPy]$^+$	2.372998	3.4	0.270708	−0.437007	−0.04985	-497.0714744
[PyHPy]$^+$	2.269724	3.3	0.266670	−0.438097	−0.05147	−497.0730063

[a]The electric potential energy $\varepsilon = {q(H)q(N^f)}/{r_2}$ associated with the N^f…H interaction.

Table S4. Geometry of BiPyH$^+$ obtained for selected r_1 distances at ωB97XD/Def2tzvp.

r_1 (N^p..H, Å)	r_2 (H..N^f, Å)	r_3 (N^p...N^f, Å)	α (N^{p}HN^f)	β (C$_4$N^{p}H)	γ (HN^{f}C$_4$′)	E Hartree
1.025207	2.024119	2.570428	110.580	175.49365	147.37505	−495.7729298
1.05	1.993658	2.564137	110.96	174.75767	148.04490	−495.7725251
1.10	1.914952	2.545255	112.339	172.84602	149.48117	−495.769708
1.15	1.788732	2.508709	115.506	169.67277	151.13609	−495.7652442
1.20	1.586534	2.440291	121.649	164.49046	153.10275	−495.7607107
1.25	1.402882	2.376221	127.106	159.68128	155.11786	−495.7580059
1.297387	1.297387	2.346111	129.423	156.83317	156.83311	−495.7573155

Table S5. Geometry of PhenH$^+$ obtained for selected r_1 distances at ωB97XD/Def2tzvp.

r_1 (N^p..H, Å)	r_2 (H..N^f, Å)	r_3 (N^p...N^f, Å)	α (N^{p}HN^f)	β (C$_4$N^{p}H)	γ (HN^{f}C$_4$′)	E Hartree
1.019898	2.226174	2.688753	105.75983	176.37667	142.50813	−572.0020078
1.05	2.203243	2.685110	105.71314	175.82143	143.23586	−572.0013643
1.10	2.156199	2.676364	105.95229	174.62459	144.47313	−571.9979315
1.15	2.089097	2.661070	106.87093	172.85628	145.77566	−571.9923753
1.20	1.983735	2.633045	109.09807	170.01137	147.17895	−571.9855869
1.25	1.735857	2.550609	116.40517	163.15824	148.88241	−571.9788076
1.25	1.732075	2.548989	116.53902	163.05713	148.89704	−571.9788116
1.30	1.423681	2.430672	126.29851	150.74148	154.37012	−571.9752034
1.332492	1.332492	2.40027	128.49316	151.81528	151.82004	−571.9747212

Table S6. Geometry of $BiPyH^+$ obtained for selected r_1 distances at ωB97XD/Def2tzvp SCRF=(Solvent=water).

r_1 (N^p..H, Å)	r_2 (H..N^f, Å)	r_3 (N^p...N^f, Å)	α (N^pHN^f)	β (C_4N^pH)	γ (HN^fC_4')	E Hartree
1.020850	2.079695	2.595704	108.82897	176.72056	146.73116	−495.8434348
1.297282	1.297282	2.343505	129.17357	156.90195	156.90195	−495.8245433

Table S7. Geometry of $PhenH^+$ obtained for selected r_1 distances at ωB97XD/Def2tzvp SCRF=(Solvent=water).

r_1 (N^p..H, Å)	r_2 (H..N^f, Å)	r_3 (N^p...N^f, Å)	α (N^pHN^f)	β (C_4N^pH)	γ (HN^fC_4')	E Hartree
1.017223	2.273055	2.710538	104.34181	177.57127	142.13853	−572.0713802
1.332127	1.332127	2.397702	128.30360	151.92158	151.92156	−572.0404339

Table S8. The geometry, energy and N^p-H stretches of $BiPyH^+$ obtained for selected r_3 distances at ωB97XD/Def2tzvp SCRF=(Solvent=water).

r_1 (N^p..H, Å)	r_2 (H..N^f, Å)	r_3 (N^p...N^f, Å)	ν(N^p-H) cm^{-1}	E Hartree
1.019401	2.080350	2.595114	3472	−495.8742696
1.021834	1.948769	2.5	3428	−495.8733569
1.023645	1.878512	2.45	3387	−495.8719693
1.027280	1.754386	2.36	3304	−495.8676032
1.027761	1.740297	2.35	3295	−495.8669472
1.296357	1.296357	2.342063	−1499	−495.8550242

Table S9. Crystallographic data for the single crystal XRD structure of $BiPyH^+ [ClO_4]^-$ obtained from acetonitrile.

Formula	$C_{20}H_{18}Cl_2N_4O_8$
$D_{calc.}$/ g cm^{-3}	1.605
μ/mm^{-1}	3.279
Formula Weight	513.293
Colour	clear colourless
Shape	prism-shaped
Size/mm^3	0.08×0.06×0.03
T/K	123.00(10)
Crystal System	monoclinic
Flack Parameter	-0.110(5)
Hooft Parameter	-0.110(5)
Space Group	*Pc*
a/Å	5.9609(1)
b/Å	12.8314(2)
c/Å	7.0548(1)
α/°	90
β/°	100.159(2)
γ/°	90
V/Å^3	531.139(15)
Z	1
Z'	0.5
Wavelength/Å	1.54184
Radiation type	Cu K_α
Θ_{min}/°	3.44
Θ_{max}/°	75.36
Measured Refl's.	18780
Indep't Refl's	2129
Refl's I≥2 σ(I)	2024
R_{int}	0.0351
Parameters	154
Restraints	2
Largest Peak	0.1584
Deepest Hole	-0.2172
GooF	1.0415
wR_2 (all data)	0.0755
wR_2	0.0744
R_1 (all data)	0.0326
R_1	0.0299

Crystal Data. $C_{20}H_{18}Cl_2N_4O_8$, M_r = 513.293, monoclinic, *Pc* (No. 7), a = 5.9609(1) Å, b = 12.8314(2) Å, c = 7.0548(1) Å, β = 100.159(2)°, $\alpha = \gamma$ = 90°, V = 531.139(15) Å^3, T = 123.00(10) K, Z = 1, Z' = 0.5, μ(Cu K_α) = 3.279, 18780 reflections measured, 2129 unique (R_{int} = 0.0351) which were used in all calculations. The final wR_2 was 0.0755 (all data) and R_1 was 0.0299 (I≥2 σ(I)).

Table S10. Crystallographic data for the single crystal XRD structure of BiPyH+ $[ClO_4]^-$ obtained from nitromethane.

Formula	$C_{10}H_9ClN_2O_4$
$D_{calc.}$/ g cm^{-3}	1.607
μ/mm^{-1}	3.282
Formula Weight	256.647
Colour	clear colourless
Shape	prism-shaped
Size/mm^3	0.27×0.17×0.15
T/K	122.97(10)
Crystal System	monoclinic
Flack Parameter	-0.227(5)
Hooft Parameter	-0.227(5)
Space Group	*Pc*
a/Å	5.9583(1)
b/Å	12.8317(2)
c/Å	7.0497(1)
α/°	90
β/°	100.174(2)
γ/°	90
V/Å^3	530.511(15)
Z	2
Z'	1
Wavelength/Å	1.54184
Radiation type	Cu K_α
Θ_{min}/°	6.90
Θ_{max}/°	66.95
Measured Refl's.	7164
Indep't Refl's	1830
Refl's I≥2 σ(I)	1826
R_{int}	0.0403
Parameters	154
Restraints	2
Largest Peak	0.1712
Deepest Hole	-0.3188
GooF	1.0589
wR_2 (all data)	0.0907
wR_2	0.0905
R_1 (all data)	0.0354
R_1	0.0353

Crystal Data. $C_{10}H_9ClN_2O_4$, M_r = 256.647, monoclinic, *Pc* (No. 7), a = 5.9583(1) Å, b = 12.8317(2) Å, c = 7.0497(1) Å, β = 100.174(2)°, α = γ = 90°, V = 530.511(15) Å^3, T = 122.97(10) K, Z = 2, Z' = 1, μ(Cu K_α) = 3.282, 7164 reflections measured, 1830 unique (R_{int} = 0.0403) which were used in all calculations. The final wR_2 was 0.0907 (all data) and R_1 was 0.0353 (I≥2 σ(I)).

Table S11. The atomic coordinates of $BiPyHClO_4$_Tq_W

7	1.588101	-1.270095	-0.530987	8	-4.132292	-1.163161	-0.814114
7	0.299588	1.045344	-0.280021	8	-1.840054	-0.915567	-0.365064
6	1.621562	1.057003	-0.036006	8	-3.347934	-1.310181	1.388146
6	2.169026	-2.471061	-0.555546	8	-3.356013	0.764414	0.261579
6	-0.452214	2.158098	-0.356939	1	1.576061	-3.274468	-0.977051
6	2.302322	-0.255281	-0.009182	1	4.111901	0.423442	0.955409
6	3.580693	-0.407617	0.514783	1	5.168031	-1.817291	0.840921
6	2.245572	2.273828	0.144877	1	3.872073	-3.710030	-0.153111
6	1.500155	3.446344	0.077390	1	-1.504850	2.000331	-0.536892
6	0.131447	3.382048	-0.173113	1	-0.471851	4.275344	-0.230676
6	4.169197	-1.666970	0.453767	1	1.985484	4.402342	0.216034
6	3.453720	-2.715049	-0.089952	1	3.308945	2.314096	0.321510
17	-3.177364	-0.646289	0.120917	1	-0.162977	0.134304	-0.399534

Table S12. The atomic coordinates of ZUTDAT_Tq_W

6	0.775527	0.122894	0.027870	6	-1.510954	1.279170	-0.035464
7	1.285342	-1.124154	-0.023601	1	-0.671707	-1.843981	0.088370
6	2.615251	-1.250903	-0.062104	1	2.997704	-2.263032	-0.109327
6	3.465782	-0.166066	-0.038155	1	4.535362	-0.324269	-0.072932
6	2.943649	1.105113	0.030536	1	3.589996	1.971676	0.056532
6	1.564409	1.262477	0.067139	1	1.126041	2.248711	0.120099
6	-0.711184	0.157761	0.023823	1	-3.007730	-2.254851	0.072227
7	-1.323182	-1.054839	0.062168	1	-4.540233	-0.250533	-0.049195
6	-2.656587	-1.235447	0.039663	1	-3.510788	2.013824	-0.109121
6	-3.468293	-0.127641	-0.027163	1	-1.063726	2.260668	-0.069474
6	-2.882606	1.135099	-0.059005				

Table S13. The atomic coordinates of ZUTDAT01_Tq_W

6	-1.506488	1.262836	0.038263	7	1.276810	-1.114534	0.022857
6	-2.870798	1.116206	0.060135	1	-1.068044	2.248874	0.072733
6	-3.444156	-0.129980	0.023972	1	-3.496683	1.996773	0.111779
6	-2.641198	-1.210708	-0.038741	1	-4.516085	-0.256896	0.042369
6	0.768284	0.119031	-0.037762	1	-2.991287	-2.231123	-0.070458
6	1.558957	1.241245	-0.065436	1	1.125370	2.230230	-0.117253
6	2.930905	1.087169	-0.031000	1	3.570117	1.959653	-0.054684
6	3.449194	-0.161829	0.040133	1	4.517925	-0.325864	0.076909
6	2.600823	-1.229015	0.061272	1	2.984468	-2.241449	0.113093
6	-0.700644	0.159439	-0.025953	1	-0.678687	-1.834551	-0.083861
7	-1.322004	-1.038611	-0.057131				

Table S14. The atomic coordinates of OPENAN_Tt_W

7	-1.837996	1.619423	-0.890499	6	5.072584	1.528709	-1.580042
7	-2.789569	-0.857443	-1.593769	6	6.220703	1.344730	-0.789742
6	-1.428442	2.834854	-0.569015	6	6.184798	0.446738	0.243488
6	-1.983352	3.597360	0.473261	6	4.975611	-0.241620	0.508266
6	-2.988635	3.062147	1.215003	6	4.862690	-1.215586	1.555269
6	-3.463477	1.768185	0.920389	6	3.859383	0.056155	-0.305773
6	-4.553185	1.166399	1.626920	1	-0.617464	3.243182	-1.166080
6	-5.023067	-0.046564	1.275052	1	-1.602983	4.592177	0.671753
6	-4.441717	-0.773522	0.184279	1	-3.441332	3.618964	2.028896
6	-4.920833	-2.036237	-0.212667	1	-4.999401	1.717848	2.447778
6	-4.337256	-2.680139	-1.263831	1	-5.855007	-0.496067	1.806540
6	-3.275290	-2.042302	-1.927300	1	-5.750644	-2.480541	0.326981
6	-2.852426	1.086515	-0.159488	1	-4.677634	-3.652678	-1.597234
6	-3.355289	-0.223367	-0.533921	1	-2.801726	-2.530067	-2.775430
7	1.512170	-0.260656	-0.787747	1	-0.486383	-0.577087	-1.133233
6	0.369816	-0.881676	-0.539614	1	-0.741236	-2.369509	0.558105
6	0.222175	-1.890868	0.428030	1	1.214173	-2.980304	1.989178
6	1.292810	-2.226896	1.212930	1	3.609295	-2.585177	2.584324
6	2.529944	-1.571868	0.997258	1	5.101983	2.211177	-2.425151
6	3.691314	-1.848283	1.792489	1	7.125004	1.893747	-1.024436
6	2.592868	-0.613470	-0.039471	1	7.057041	0.256520	0.858989
7	3.917790	0.922525	-1.353768	1	5.739180	-1.425492	2.158890

Table S15. The atomic coordinates of OPENAN01_Tt_W

7	-1.837996	1.619423	-0.890499	6	5.072584	1.528709	-1.580042
7	-2.789569	-0.857443	-1.593769	6	6.220703	1.344730	-0.789742
6	-1.428442	2.834854	-0.569015	6	6.184798	0.446738	0.243488
6	-1.983352	3.597360	0.473261	6	4.975611	-0.241620	0.508266
6	-2.988635	3.062147	1.215003	6	4.862690	-1.215586	1.555269
6	-3.463477	1.768185	0.920389	6	3.859383	0.056155	-0.305773
6	-4.553185	1.166399	1.626920	1	-0.617464	3.243182	-1.166080
6	-5.023067	-0.046564	1.275052	1	-1.602983	4.592177	0.671753
6	-4.441717	-0.773522	0.184279	1	-3.441332	3.618964	2.028896
6	-4.920833	-2.036237	-0.212667	1	-4.999401	1.717848	2.447778
6	-4.337256	-2.680139	-1.263831	1	-5.855007	-0.496067	1.806540
6	-3.275290	-2.042302	-1.927300	1	-5.750644	-2.480541	0.326981
6	-2.852426	1.086515	-0.159488	1	-4.677634	-3.652678	-1.597234
6	-3.355289	-0.223367	-0.533921	1	-2.801726	-2.530067	-2.775430
7	1.512170	-0.260656	-0.787747	1	-0.486383	-0.577087	-1.133233
6	0.369816	-0.881676	-0.539614	1	-0.741236	-2.369509	0.558105
6	0.222175	-1.890868	0.428030	1	1.214173	-2.980304	1.989178
6	1.292810	-2.226896	1.212930	1	3.609295	-2.585177	2.584324
6	2.529944	-1.571868	0.997258	1	5.101983	2.211177	-2.425151
6	3.691314	-1.848283	1.792489	1	7.125004	1.893747	-1.024436
6	2.592868	-0.613470	-0.039471	1	7.057041	0.256520	0.858989
7	3.917790	0.922525	-1.353768	1	5.739180	-1.425492	2.158890

Table S16. The atomic coordinates of ZUTDEX_Tq_W

7	-1.327607	-1.542925	-0.069028	6	-1.457857	0.900724	0.004334
6	-2.647315	-1.576902	-0.026588	6	1.401182	0.934829	-0.022330
6	-3.447904	-0.418118	0.030767	1	0.799948	-2.303652	0.056450
6	-2.859086	0.805038	0.027315	1	-3.108729	-2.557159	-0.049015
6	-0.708294	2.129186	0.024828	1	-4.523897	-0.520985	0.061086
6	0.635237	2.148998	-0.009365	1	-3.453998	1.709534	0.053382
6	2.804160	0.851793	-0.034581	1	-1.261274	3.059968	0.051892
6	3.452288	-0.358873	-0.002418	1	1.171753	3.088677	-0.008686
6	2.715885	-1.528043	0.053831	1	3.381608	1.767250	-0.059293
7	1.375901	-1.462414	0.037811	1	4.529977	-0.416810	-0.003211
6	0.687708	-0.283549	-0.019035	1	3.150967	-2.513920	0.102049
6	-0.746740	-0.317671	-0.024447				
